# Enhanced Sensitivity and Altered EEG Patterns During General Anesthesia in BTBR Mice, a Model of Autism

**DOI:** 10.3390/brainsci16040391

**Published:** 2026-04-01

**Authors:** Yeonsu Kim, Seounghun Lee, Seong-Eun Kim, Yeojung Kim, Xianshu Ju, Yulim Lee, Tao Zhang, Juyeon Kim, Sungho Choi, Jun Young Heo, Woosuk Chung, Jiho Park

**Affiliations:** 1 Biomedical Research Institute, Chungnam National University Hospital, Daejeon 35015, Republic of Korea; 2 Department of Anesthesiology and Pain Medicine, Chungnam National University Sejong Hospital, 20, Bodeum 7-ro, Sejong 30099, Republic of Korea; 3 Department of Anesthesiology and Pain Medicine, Chungnam National University School of Medicine, Daejeon 35015, Republic of Korea; 4 Department of Applied Artificial Intelligence, Seoul National University of Science and Technology, Seoul 01811, Republic of Korea; 5 Department of Medical Science, Chungnam National University School of Medicine, Daejeon 35015, Republic of Korea; 6 Department of Biochemistry, Chungnam National University School of Medicine, Daejeon 35015, Republic of Korea; 7 Department of Anesthesiology and Pain Medicine, Chungnam National University Hospital, Daejeon 35015, Republic of Korea

**Keywords:** autism spectrum disorder, sevoflurane, electroencephalography, BTBR mice, general anesthesia, burst suppression

## Abstract

**Highlights:**

**What are the main findings?**

- BTBR mice show increased sensitivity to sevoflurane anesthesia compared with C57BL/6J mice.
- EEG analysis demonstrates earlier burst suppression and anteriorization of theta power in BTBR mice.

**What are the implications of the main findings?**

- These findings suggest altered EEG and cortical response patterns to volatile anesthetics in an autism mouse model.
- The results support further investigation into whether EEG-based anesthetic monitoring may be useful in ASD-relevant settings.

**Abstract:**

Background/Objectives: Alterations in excitation/inhibition (E/I) balance, involving both inhibitory and excitatory signaling, have been implicated in the pathophysiology of autism spectrum disorder (ASD). Volatile anesthetics, including sevoflurane, act on multiple molecular and network targets, and anesthetic sensitivity may therefore differ in ASD. This study investigated whether sevoflurane sensitivity is altered in BTBR T+Itpr3tf/J (BTBR) mice, a widely used mouse model of ASD. Methods: Sevoflurane sensitivity was compared between BTBR mice and C57BL/6J (B6) control mice using behavioral and electroencephalographic (EEG) analyses. The minimum alveolar concentration required to abolish nociceptive responses (MAC*sevo*) and the sevoflurane concentration associated with recovery of the righting reflex (RR*sevo*) were measured. Dose-dependent EEG changes, including burst suppression and theta power distribution, were also evaluated. Results: MAC*sevo* did not differ significantly between BTBR and B6 mice. However, RR*sevo* was significantly lower in BTBR mice (1.10 ± 0.10%) compared with B6 mice (1.65 ± 0.13%; *p* < 0.001). EEG analyses demonstrated that burst suppression occurred at lower sevoflurane concentrations in BTBR mice (2.0%) than in B6 mice (2.4%). In addition, topographical mapping revealed distinct theta power dynamics between the two strains during anesthesia. Conclusions: BTBR mice exhibit increased sensitivity to sevoflurane during emergence from anesthesia and show distinct EEG patterns compared with control mice. These findings suggest altered anesthetic responsiveness in a mouse model of ASD and support the possibility that network-level neurophysiological differences may influence anesthetic responses. Further studies are needed to clarify whether similar alterations are present across other ASD models and human ASD populations.

## 1. Introduction

Autism spectrum disorder (ASD) is a complex genetic neurodevelopmental disorder characterized by clinical symptoms of impaired sociability and restrictive repetitive behaviors [1]. Recent epidemiological studies indicate that the prevalence of ASD has continued to increase, with current estimates approaching approximately 1 in 31 children (~3.2%) in the most recent surveillance data [2]. Given this growing prevalence, anesthesiologists and other healthcare providers are increasingly likely to encounter patients with ASD in a perioperative setting. This can be a significant challenge, as these patients display a wide variety of symptoms that may cause significant difficulties [3,4,5]. Although several perioperative management strategies have been suggested, including appropriate premedication and minimizing environmental stressors [6,7,8], most of these recommendations focus on pre- and postoperative periods. In contrast, intraoperative anesthetic management has received less attention, despite its critical importance in ensuring patient safety [9,10]. Recent clinical studies suggest that patients with ASD may exhibit differences in perioperative behavior, emergence characteristics, and anesthetic responses, although the underlying mechanisms remain incompletely understood [11].

Although the precise mechanism underlying ASD has not been fully elucidated, many studies have implicated an excitation/inhibition (E/I) imbalance as playing a key role [12,13]. Several clinical studies have reported a reduction in γ-aminobutyric acid (GABA) in diverse brain regions [14,15]. The importance of changes in GABAergic synaptic transmission is further supported by animal models of autism, which show that modulation of GABAergic synaptic transmission can ameliorate behavioral deficits [16,17,18]. One such animal model is the BTBR T^+^Itpr3^tf^/J (hereafter, BTBR) mouse, an inbred strain widely studied as an animal model of autism [19]. Previous studies have reported reduced GABAergic synaptic transmission in various brain regions of BTBR mice [16,18]. Because volatile anesthetics such as sevoflurane act on multiple molecular and network targets, including GABA_A_ receptor-mediated inhibition as well as excitatory and thalamocortical network processes, anesthetic sensitivity in ASD models may reflect broader circuit-level differences rather than a single neurotransmitter mechanism [20,21,22]. Sevoflurane is one of the most widely used volatile anesthetics in clinical practice due to its rapid onset, favorable pharmacokinetics, and frequent use in pediatric and neurodevelopmental populations. Nevertheless, few studies have systematically characterized the acute neurophysiological and behavioral sensitivity to sevoflurane in the specific context of ASD.

Given these findings, we hypothesized that BTBR mice would exhibit altered sensitivity to sevoflurane, reflecting differences in neural circuit function and excitation/inhibition balance rather than a purely GABAergic mechanism. To test this hypothesis, we compared behavioral and electroencephalographic (EEG) responses to sevoflurane between BTBR mice, a model of ASD, and C57BL/6J (B6) mice. Sevoflurane sensitivity was assessed by determining the minimum alveolar concentration (MAC*sevo*) and the righting reflex recovery concentration (RR*sevo*), together with an EEG-based analysis of dose-dependent burst suppression and cortical power dynamics.

## 2. Materials and Methods

### 2.1. Animals

All experimental procedures were approved by the Committee of Animal Research at Chungnam National University (Daejeon, Republic of Korea; 202109A-CNU-178). BTBR mice, purchased from Jackson Laboratory (Bar Harbor, ME, USA), and C57BL/6 mice, purchased from Damul Science (Daejeon, Korea), were housed in a controlled environment (temperature: 22 °C; humidity: 40%) under a 12 h reverse light/dark cycle with free access to food and water. All mice were group-housed (4–5 animals per individually ventilated cage) with standard bedding and nesting material, and environmental enrichment was provided. All mice used in this study were male and 8–10 weeks old. After experiments, mice were euthanized using carbon dioxide (CO_2_) inhalation.

### 2.2. Measurement of MACsevo

**Figure 1:**
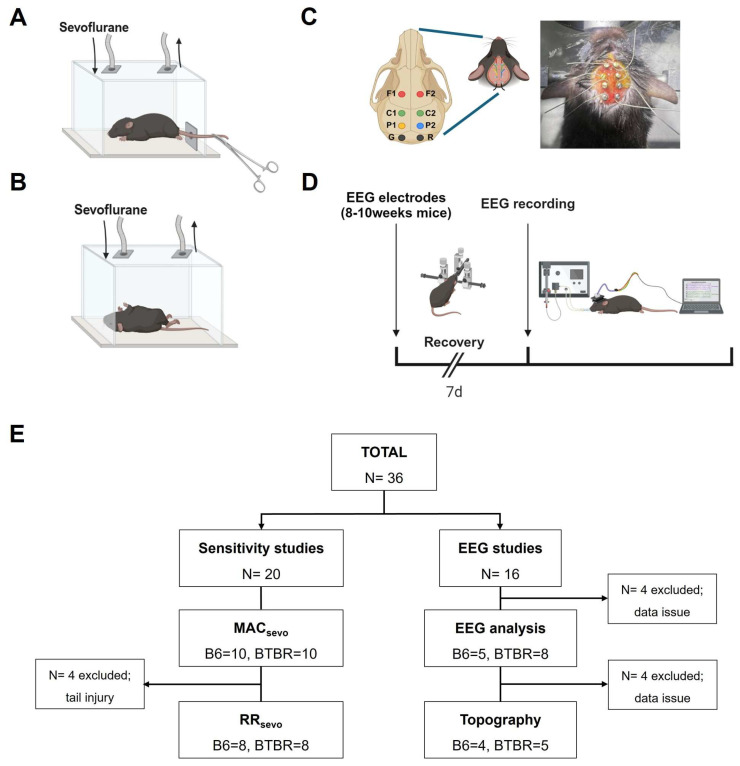
Schematic illustration of experimental design and flow chart. (**A**) Procedure for determining MAC*sevo* using tail-clamp stimulation. (**B**) Procedure for measuring RR*sevo*, the concentration of sevoflurane required for recovery of the righting reflex. (**C**) Schematic and representative image of screw electrode placement on the skull of BTBR mice. Electrodes were positioned over the bilateral prefrontal and parietal cortices, with additional screws over the cerebellum serving as reference and ground. Animals were anesthetized with sevoflurane and secured in a stereotaxic frame during implantation. (**D**) EEG electrodes were implanted in adult mice (8–10 weeks old), followed by EEG recordings conducted 1 week after implantation. (**E**) Study design flow chart. Abbreviations: MAC*sevo*, minimum alveolar concentration for sevoflurane; RR*sevo*, sevoflurane concentration required to achieve recovery of righting reflex; EEG, electroencephalogram; con, control; sevo, sevoflurane.

The MAC of sevoflurane, defined as the lowest concentration that prevents movement in response to a painful stimulus, was measured as previously described [23]. Briefly, B6 and BTBR mice were placed in a gas-tight sevoflurane anesthesia chamber (19.5 cm in length, 12 cm in width, 7 cm in height) with their tail protruding out of the chamber to provide access for delivery of a tail clamp stimulus. Mouse body temperature was maintained by a heating pad (37 °C), placed under the anesthesia chamber. Sevoflurane (Ilsung, Seoul, Republic of Korea) and CO_2_ concentrations were continuously measured using a gas monitor (Dräger Vamos 2; Draegerwerk AG & Co., Lübeck, Germany). The initial volatile sevoflurane concentration was 2.5%, delivered at a rate of 5 L/min at 36 °C using an AnaPod Humi-Therm Heated Humidification System (Westmed, Inc., Tucson, AZ, USA), yielding an inspired oxygen fraction [FiO_2_] of 0.4. An initial tail-clamp stimulus was applied to the middle third of the tail after a 10 min sevoflurane exposure using a 15 cm hemostatic forceps, and mouse movements were monitored (Figure 1A). A positive response was defined as gross motor activities of the head, body, extremities or all three after tail clamping [24]. The concentration of anesthetic was increased by 0.1% until the positive response disappeared. Tail clamping was performed at the end of each 10 min interval of sevoflurane exposure.

### 2.3. Measurement of RRsevo

RR*sevo*, defined as the sevoflurane concentration at which a mouse regained the ability to right itself from the supine to the prone position, was determined in a quiet environment as previously described [23] (Figure 1B). After a 20 min exposure to 2.0% sevoflurane in the anesthesia chamber, mice were placed in a supine position, and the concentration of sevoflurane was decreased gradually in 0.1% decrements over a 10 min interval until a sevoflurane concentration that allowed them to return to the prone position was reached.

### 2.4. EEG Electrode Implantation and Recording

Mice were continuously anesthetized with 2.0% sevoflurane during electrode implantation. Mice were fixed in a stereotaxic apparatus (Model 940; David KOPF Instruments, Tujunga, CA, USA) and analgesia was provided by subcutaneously injecting 0.5 mL of 1% lidocaine (Dai Han Pharm. Co., Ltd., Seoul, Republic of Korea) before making an incision in the skin. Eight EEG stainless-steel screws (0.10” Mouse EEG Screw with Wire lead; Pinnacle Technology, Lawrence, KS, USA) were surgically implanted on the skull as follows: two for the bilateral prefrontal cortex (+1.8 mm anterior-posterior [AP], ±1.0 mm medial-lateral [ML]); four for the bilateral parietalis (−1.4 mm AP, ±1.0 mm ML and −2.8 mm AP, ±1.0 mm ML); and two placed over the cerebellum that served as reference or ground screws [25,26] (Figure 1C). The leads connecting the screws were soldered to an 8-pin surface mount (8-pin connector for mice, 90-degree pin configuration; Pinnacle Technology, Lawrence, KS, USA), and screws and the head mount were fixed by applying dental cement (Vertex Self-Curing; Vertex-Dental B.V., Soesterberg, The Netherlands). Body temperature, measured throughout the procedure, was maintained at 37 °C using a heating pad (JD-OT-06DT; Jeung Do Bio& Plant Co., Ltd., Wonju, Republic of Korea). Mice were returned to their home cages after confirming full recovery.

One week after electrode implantation, mice were placed in a quiet environment for EEG recording. After confirmation of loss of consciousness in the anesthesia chamber (sevoflurane, 3.0%), mice were connected to a ventilator (Model 845 MiniVent; Hugo Sachs Elektronik, March-Hugstetten, Germany) via a conical-shaped, custom-made facemask (Figure 1D). Ventilator settings (tidal volume = weight [kg] × 10 mL; respiration rate = 180 bpm) were modified from a previous study [27]. The initial sevoflurane concentration was 2.0% (FiO_2_, 0.4; fresh gas flow = 5 L/min). EEG recording was initiated after a 10 min exposure, and the concentration of sevoflurane was gradually increased by 0.2% to a maximum of 2.8%. EEG was recorded for 5 min after each increment, starting when the sevoflurane concentration stabilized. Data were acquired using Sirenia Acquisition 2.2.2 software (Pinnacle Technology). Ventilator settings, inspired oxygen fraction, fresh gas flow, sevoflurane concentration increments, and body temperature were standardized across animals during EEG recordings.

### 2.5. EEG Analysis, Burst-Suppression Quantification, and Topographical Mapping

EEG spectrograms were generated using the multitaper spectral estimation method implemented in the Chronux toolbox (version 2.1.2) [28] and custom MATLAB scripts (MATLAB, version R2023b; MathWorks, Natick, MA, USA). Signals were referenced to the frontal electrode. Power spectra were calculated in consecutive 2-s windows without overlap using a time–bandwidth product of three and five tapers. For each group, median power values across all animals were used to construct group-averaged spectrograms.

Suppression was defined as an EEG signal with an amplitude < 5 mV lasting longer than 0.5 s, consistent with previously established criteria for identifying burst suppression patterns in rodent EEG studies [29]. Suppression events were manually identified from raw EEG signals based on visual inspection during anesthesia. EEGs were segmented into bursts and suppressions using a voltage-based threshold. The burst-suppression ratio (BSR) was calculated as the percentage of time spent in suppression for each recording [30].

Topographical maps of EEG power in the theta frequency range (4–8 Hz) were generated using the FieldTrip toolbox [31]. The median spectral power values calculated from each channel were referenced across scalp locations based on standard mouse EEG electrode positions [32]. These maps illustrate how theta power was distributed across different brain regions during anesthesia, before the onset of burst suppression. Group-averaged maps were then produced by averaging the median theta power across all animals in each group to allow visual comparison of spatial activity patterns.

### 2.6. Study Design

The study was conducted using two independent cohorts of mice. In the first cohort, sevoflurane sensitivity was evaluated by measuring the MAC*sevo* and RR*sevo*. In the second cohort, anesthetic-induced changes in brain activity were assessed by obtaining EEG recordings (Figure 1E).

A total of 36 mice were used in this study. For the sevoflurane sensitivity experiments, 20 mice were initially included (B6 = 10, BTBR = 10). Four BTBR mice were excluded from RR*sevo* analysis due to tail injury, resulting in final group sizes of B6 = 8 and BTBR = 8. For EEG experiments, 16 mice were enrolled (B6 = 8, BTBR = 8). Four animals were excluded because of data quality issues (excessive noise or electrode detachment), and the remaining mice were included in the final analyses: B6 = 5 and BTBR = 8 for burst-suppression quantification, and B6 = 4 and BTBR = 5 for topographical theta-band mapping. These exclusion criteria were defined a priori, and no additional animals were removed.

These exclusions were based on predefined technical or quality-control criteria and were not related to the observed experimental outcomes, thereby reducing the likelihood of systematic selection bias.

Animals were randomly assigned to each experimental group. Behavioral scoring, including determination of the righting reflex, and subsequent EEG suppression analyses were performed by investigators blinded to the group identity. Sample sizes were determined based on previous studies using similar experimental endpoints and practical considerations related to technically demanding EEG recordings in anesthetized mice. Although a formal a priori power calculation was not performed, the sample sizes are similar to those used in prior preclinical EEG studies under anesthesia or in mouse EEG datasets [29,30,32].

### 2.7. Statistical Analysis

Statistical analyses were carried out using GraphPad Prism version 8.3 software (GraphPad Inc., La Jolla, CA, USA). All continuous variables were tested for normality and homogeneity of variance. Student’s *t*-test was performed if both cases were met, whereas a Mann–Whitney test was performed in cases where one of the conditions was not met. Data are expressed as means ± standard deviation (SD), and statistical significance was set at *p* < 0.05.

## 3. Results

### 3.1. Differential Behavioral Sensitivity to Sevoflurane in BTBR and B6 Mice

**Figure 2:**
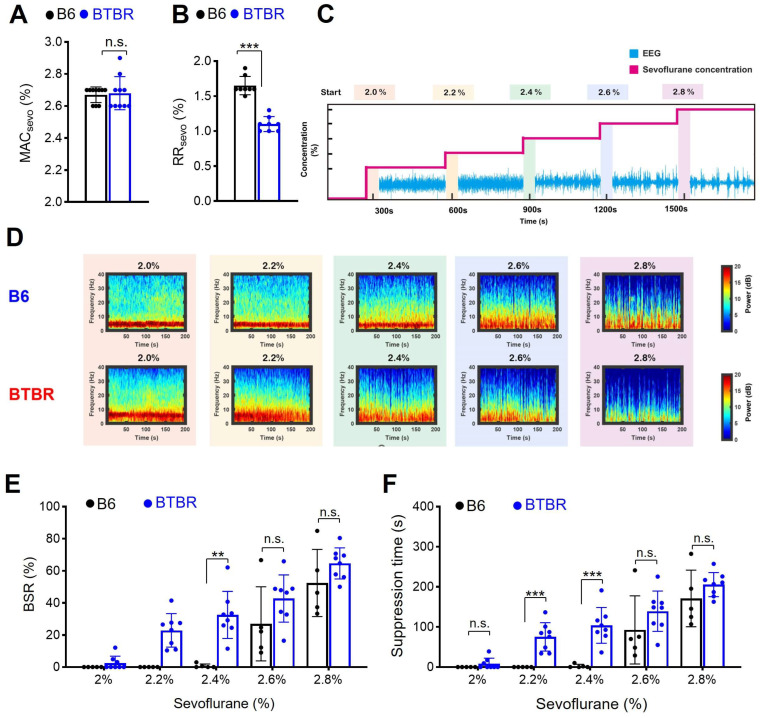
BTBR mice are more susceptible to sevoflurane-induced burst suppression. (**A**) MAC*sevo* was comparable between B6 and BTBR mice (n = 10/group; *p* = 0.785, Student’s *t*-test). (**B**) RR*sevo* was significantly decreased in BTBR mice compared to B6 mice (n = 8/group; *p* < 0.001, Student’s *t*-test). (**C**) Sequential increase of sevoflurane concentration during EEG recordings. (**D**) Median EEG power values for each group, converted into density spectral array (DSA) representations. (**E**) BSR, measured during sevoflurane anesthesia for B6 (n = 5) and BTBR (n = 8) mice (sevoflurane 2.2%, *p* = 0.003, Mann–Whitney test; sevoflurane 2.4%, *p* = 0.003, Mann–Whitney test). (**F**) Suppression time for each sevoflurane concentration, compared between B6 (n = 5) and BTBR (n = 8) mice (2.2%, *p* < 0.001, Mann–Whitney test; 2.4%, *p* < 0.001, Mann–Whitney test). Values are presented as means ± SD (n.s., not significant, ** *p* < 0.01, *** *p* < 0.001). MAC*sevo*, minimum alveolar concentration for sevoflurane; RR*sevo*_,_ concentration required to achieve recovery of righting reflex; EEG, electroencephalogram; BSR, burst suppression ratio.

To compare the sensitivity to sevoflurane between BTBR and B6 mice, we determined MAC*sevo*, defined as the sevoflurane concentration required to prevent movement in response to a noxious tail-clamp stimulus. MAC*sevo* values were comparable for B6 (2.67% ± 0.05%) and BTBR (2.68% ± 0.10%) mice (Figure 2A). To further evaluate anesthetic sensitivity at the supraspinal level associated with loss and recovery of consciousness, we measured RR*sevo*, which corresponds to the minimum alveolar concentration at awakening (MAC*awake*)—that is, the concentration of sevoflurane at which there are signs of regaining consciousness. BTBR mice regained their righting reflex at a significantly lower sevoflurane concentration (1.10% ± 0.10%) compared to B6 mice (1.65% ± 0.13%; Figure 2B). These findings indicate that, whereas immobilizing sensitivity (MAC*sevo*) was comparable between strains, BTBR mice recovered the righting reflex at a lower sevoflurane concentration. Because MAC*sevo* and RR*sevo* reflect distinct physiological endpoints—spinal immobility and supraspinal recovery, respectively—this difference should be interpreted in that context.

### 3.2. Sevoflurane-Induced Burst Suppression Appears at a Lower Sevoflurane Concentration in BTBR Mice

To further evaluate differences in supraspinal anesthetic responsiveness to sevoflurane in BTBR mice, we evaluated the occurrence of burst suppression by recording frontal EEGs while gradually increasing the sevoflurane concentration (Figure 2C) [33,34]. Burst suppression, an EEG pattern consisting of high-voltage activity alternating with isoelectric quiescence, represents a state of deep anesthesia [33]. Burst suppression appeared at a significantly lower sevoflurane concentration in BTBR mice than in B6 mice. EEG suppression was evident at 2.0% sevoflurane in BTBR mice, whereas it first appeared at 2.4% in B6 mice (Figure 2D). Furthermore, at 2.2% and 2.4% sevoflurane, both the burst suppression ratio (BSR) and suppression time were significantly higher in BTBR mice than in B6 mice (*p* < 0.05; Figure 2E,F). These EEG findings further support the possibility that BTBR mice exhibit altered cortical responsiveness to the anesthetic effects of sevoflurane compared with B6 mice.

### 3.3. Rapid Anterior Theta Shift in BTBR Mice Compared to B6 Mice During Sevoflurane Anesthesia

**Figure 3:**
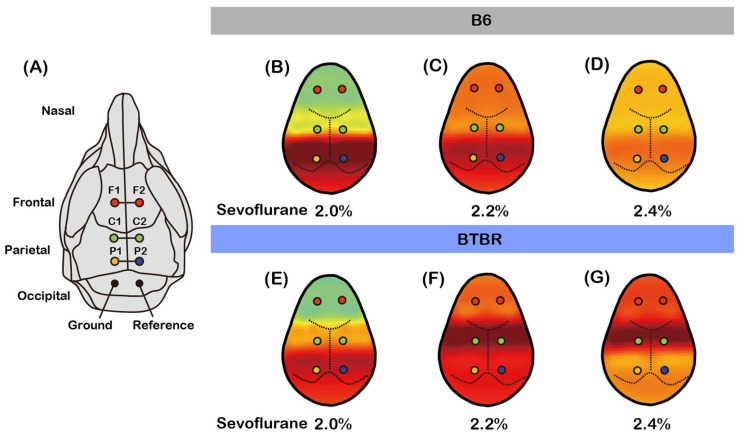
Topographical distribution of theta power during sevoflurane anesthesia in B6 and BTBR mice. (**A**) Schematic diagram of electrode placement on the mouse scalp, indicating the frontal (F1, F2), central (C1, C2), and parietal (P1, P2) channels used for EEG recording. Ground and reference electrodes are positioned in the occipital region. (**B**–**D**) Topographical maps of theta power (4–8 Hz) in B6 mice during sevoflurane anesthesia at concentrations of 2.0–2.4%. At 2.0%, theta power was primarily localized to parietal regions (P1, P2). As the concentration increased to 2.2%, there was a slight anterior shift towards central channels (C1, C2), with minimal progression towards frontal channels (F1, F2) at 2.4%. (**E**–**G**) Topographical maps of theta power in BTBR mice during sevoflurane anesthesia at concentrations of 2.0–2.4%. Similar to B6 mice, theta power was initially localized to parietal regions (P1, P2) at 2.0%. However, as the sevoflurane concentration increased to 2.2%, there was a more pronounced anterior shift of theta power towards central channels (C1, C2), with a further shift to frontal channels (F1, F2) at 2.4%.

Theta oscillations (4–8 Hz) observed during general anesthesia are thought to reflect an intermediate stage of consciousness suppression, likely mediated by enhanced GABAergic inhibition, that slows cortico-hippocampal communication and interferes with cognitive integration [35,36,37]. The topographical distribution of theta power during sevoflurane anesthesia revealed significant differences in the progression of theta activity between B6 and BTBR mice (Figure 3). EEG topography was constructed based on the standard electrode configuration used for mouse cortical recordings (Figure 3A). Initially, theta power was predominantly localized to the parietal regions of both groups at 2.0% sevoflurane (Figure 3B,C). This distribution was concentrated within P1 and P2 channels. As the sevoflurane concentration increased to 2.2%, a notable divergence in theta power distribution between the groups emerged. B6 mice exhibited only a slight shift of theta power toward the central channels, maintaining a significant presence in the parietal region (Figure 3D). In contrast, BTBR mice exhibited a pronounced anterior shift of theta power from occipital regions to the central channels (C1, C2) (Figure 3E). At 2.4% sevoflurane, the differences between the groups become even more evident. B6 mice showed slower progression, with theta power remaining minimal in the frontal regions (Figure 3F). In contrast, BTBR mice exhibited a significant anterior shift of theta power, which was prominently observed in the frontal channels (F1, F2) (Figure 3G). These findings indicate that theta oscillations shift toward the frontal cortex more rapidly in BTBR mice than in B6 mice as anesthesia depth increases, suggesting altered cortical dynamics during sevoflurane anesthesia.

## 4. Discussion

In the present study, we examined anesthetic sensitivity at the supraspinal level using EEG-based cortical recordings and identified distinct oscillatory patterns during sevoflurane anesthesia in BTBR mice, a well-established model of ASD. Compared with C57BL/6J (B6) mice, BTBR mice exhibited altered sensitivity to sevoflurane, particularly during emergence from anesthesia, requiring a lower anesthetic concentration for righting reflex recovery. EEG analyses further demonstrated more frequent burst suppression and a pronounced anterior shift of theta power at lower anesthetic concentrations. In this context, anesthesia was used primarily as a physiological probe to evaluate differences in neural responsiveness between BTBR mice and control mice.

An imbalance between excitatory and inhibitory neurotransmission is a key pathophysiological feature of ASD. Because many anesthetics act as positive allosteric modulators of the GABA_A_ receptor, altered GABAergic signaling in ASD may influence anesthetic sensitivity. However, these findings should not be interpreted as evidence for a purely GABAergic mechanism. ASD is biologically heterogeneous, and altered anesthetic sensitivity in BTBR mice may arise from interacting changes in inhibitory signaling, excitatory transmission, and large-scale network dynamics [12,13]. Previous studies using autism-related mouse models, such as *Fmr1* and *Shank3* mutants, have reported abnormal behavioral responses to anesthetics; however, these studies primarily focused on behavioral or spinal endpoints without directly assessing cortical mechanisms [23,38,39]. The present study provides neurophysiological evidence of altered EEG responses to sevoflurane in BTBR mice and establishes an EEG-based framework for characterizing anesthetic responses in autism models. MAC, based on movement during a noxious supramaximal stimulus, has been used as the standard measurement for evaluating the potency of anesthetics [40]. In measuring MAC*sevo* by application of tail-clamping, we initially discovered that MAC*sevo* values were comparable between BTBR and B6 mice. However, MAC reflects immobility, which is mediated primarily at the spinal cord level [41,42]. Thus, MAC is not suitable for assessing anesthetic effects on unconsciousness or amnesia, which depend on supraspinal mechanisms [43]. For this purpose, previous studies used the righting-reflex in rodents, which correlates with MAC*awake* in humans [21]. In our experiments, BTBR mice regained the righting reflex at lower concentrations of sevoflurane compared with B6 mice, suggesting altered anesthetic responsiveness at the supraspinal level. However, this interpretation remains indirect, and additional measures of consciousness would be needed to establish supraspinal mechanisms more definitively.

To complement these behavioral findings, we next examined EEG changes during sevoflurane exposure [34]. Our EEG results showed that burst suppression, a pattern of brain waves that emerges at excessive depths of anesthesia [44], occurred at lower sevoflurane concentrations, strongly suggesting that BTBR mice are indeed more sensitive to sevoflurane. Sevoflurane’s actions extend beyond potentiating GABA_A_ receptor-mediated inhibition to include inhibition of NMDA receptor-mediated excitation, activation of background potassium channels, and suppression of voltage-gated sodium channels [20,21,22]. These mechanisms can reduce cortical excitability and, within thalamocortical networks, facilitate earlier transitions to synchronized low-frequency activity and burst suppression. Because sevoflurane acts on multiple molecular targets, the present findings cannot determine the precise synaptic mechanisms underlying the altered anesthetic sensitivity observed in BTBR mice. Accordingly, the present study should be interpreted as demonstrating altered neurophysiological responsiveness rather than identifying a specific molecular mechanism or signaling pathway.

Enhanced sensitivity was further confirmed by a topographical analysis of EEG, which revealed a more rapid progression of the anteriorization of theta power in BTBR mice. Beyond burst suppression, changes in theta activity further point to altered cortical processing in BTBR mice. The anterior shift of theta power appears to reflect an imbalance in thalamocortical modulation rather than a simple redistribution of oscillatory power [45]. Under normal conditions, posterior theta activity is associated with sensory integration, whereas frontal theta becomes dominant when cortical top-down control increases [46,47]. The earlier emergence of frontal theta power in BTBR mice may therefore indicate an abnormal transition in cortical state regulation during anesthesia [48]. A previous study on propofol-induced unconsciousness demonstrated that anesthetics alter thalamocortical oscillations, resulting in the anteriorization of alpha rhythms within prefrontal–thalamic circuits [49]. Although our study focused on theta oscillations, a similar anteriorization pattern observed under sevoflurane anesthesia may reflect comparable contributions of thalamocortical mechanisms to altered cortical synchronization in BTBR mice.

This study has several limitations. The relatively small sample size in the EEG analyses may reduce statistical power, increase susceptibility to random effects, and limit the detection of more subtle or complex EEG features. In addition, several physiological variables that may influence EEG activity and anesthetic depth, including CO_2_ levels, ventilation, and metabolic parameters, were not continuously monitored throughout all experimental conditions. Although major experimental settings were standardized across groups, these factors may have contributed to variability. A further limitation is the absence of a direct assessment of how the increased anesthetic sensitivity observed in BTBR mice might influence postoperative outcomes. The clinical significance of the lower burst suppression thresholds observed in BTBR mice should therefore be interpreted with caution. Although the evidence remains inconclusive, intraoperative burst suppression has been discussed as a potential marker associated with adverse postoperative cognitive outcomes, including postoperative delirium (POD) [50,51]. In this context, our findings may provide a physiological basis for future translational studies investigating whether individualized anesthetic strategies or EEG-guided monitoring may be beneficial in patients with ASD. It should also be noted that reduced GABAergic transmission is not the sole mechanism proposed for E/I imbalance in autism [12]. Alterations in excitatory synaptic transmission have also been described in both patients and animal models [12,52]. Therefore, anesthetic sensitivity may differ depending on the underlying neurobiological mechanisms across ASD subtypes.

Another limitation of this study is that the findings were obtained from a single ASD mouse model (BTBR mice). Although BTBR mice reproduce several behavioral and neurobiological features of ASD, they cannot represent the full heterogeneity of autism spectrum disorders. Therefore, caution is warranted when extrapolating these findings to the broader ASD population. Consistent with this interpretation, a recent study employing an autism mouse model with reduced glutamatergic transmission also reported increased sensitivity to isoflurane [23], suggesting the need for further research to understand how distinct neurobiological alterations across ASD subtypes may differentially influence anesthetic sensitivity.

## 5. Conclusions

Taken together, our findings indicate that sensitivity to sevoflurane is increased in BTBR mice, a widely used mouse model showing ASD-like behavioral and neurophysiological features. These results support altered anesthetic responsiveness in this model, but they should not be directly generalized to the broader ASD population. Future studies are needed to determine whether similar alterations occur across diverse ASD populations and to elucidate the underlying neurophysiological mechanisms, including the specific synaptic and network processes contributing to these differences in anesthetic response.

## Data Availability

The minimal dataset supporting the findings of this study is included in the Article. Additional raw EEG datasets are available from the corresponding author upon reasonable request due to the large size of the raw data files.

## References

[1] Association A.P. (2013). DSM-5 Diagnostic Classification. Diagnostic and Statistical Manual of Mental Disorders.

[2] Shaw K.A., Williams S., Patrick M.E., Valencia-Prado M., Durkin M.S., Howerton E.M., Ladd-Acosta C.M., Pas E.T., Bakian A.V., Bartholomew P. (2025). Prevalence and Early Identification of Autism Spectrum Disorder Among Children Aged 4 and 8 Years—Autism and Developmental Disabilities Monitoring Network, 16 Sites, United States, 2022. MMWR Surveill. Summ..

[3] Grove J., Ripke S., Als T.D., Mattheisen M., Walters R.K., Won H., Pallesen J., Agerbo E., Andreassen O.A., Anney R. (2019). Identification of common genetic risk variants for autism spectrum disorder. Nat. Genet..

[4] Sharma S.R., Gonda X., Tarazi F.I. (2018). Autism Spectrum Disorder: Classification, diagnosis and therapy. Pharmacol. Ther..

[5] Riquelme I., Hatem S.M., Montoya P. (2016). Abnormal Pressure Pain, Touch Sensitivity, Proprioception, and Manual Dexterity in Children with Autism Spectrum Disorders. Neural Plast..

[6] Vlassakova B.G., Emmanouil D.E. (2016). Perioperative considerations in children with autism spectrum disorder. Curr. Opin. Anaesthesiol..

[7] Taghizadeh N., Heard G., Davidson A., Williams K., Story D. (2019). The experiences of children with autism spectrum disorder, their caregivers and health care providers during day procedure: A mixed methods study. Paediatr. Anaesth..

[8] Reddy S., Deutsch N. (2020). Behavioral and Emotional Disorders in Children and Their Anesthetic Implications. Children.

[9] O’Brien E.M., Stricker P.A., Harris K.A., Liu H., Griffis H., Muhly W.T. (2024). Perioperative Management and Outcomes in Patients with Autism Spectrum Disorder: A Retrospective Cohort Study. Anesth. Analg..

[10] Brown S., Rabenstein K., Doherty M. (2024). Autism and anaesthesia: A simple framework for everyday practice. BJA Educ..

[11] Whippey A., Bernstein L.M., O’Rourke D., Reddy D. (2019). Enhanced perioperative management of children with autism: A pilot study. Can. J. Anesth./J. Can. D’anesthésie.

[12] Lee E., Lee J., Kim E. (2017). Excitation/Inhibition Imbalance in Animal Models of Autism Spectrum Disorders. Biol. Psychiatry.

[13] Port R.G., Oberman L.M., Roberts T.P. (2019). Revisiting the excitation/inhibition imbalance hypothesis of ASD through a clinical lens. Br. J. Radiol..

[14] Gaetz W., Bloy L., Wang D.J., Port R.G., Blaskey L., Levy S.E., Roberts T.P. (2014). GABA estimation in the brains of children on the autism spectrum: Measurement precision and regional cortical variation. Neuroimage.

[15] Puts N.A.J., Wodka E.L., Harris A.D., Crocetti D., Tommerdahl M., Mostofsky S.H., Edden R.A.E. (2017). Reduced GABA and altered somatosensory function in children with autism spectrum disorder. Autism Res..

[16] Han S., Tai C., Jones C.J., Scheuer T., Catterall W.A. (2014). Enhancement of inhibitory neurotransmission by GABAA receptors having alpha2,3-subunits ameliorates behavioral deficits in a mouse model of autism. Neuron.

[17] Han S., Tai C., Westenbroek R.E., Yu F.H., Cheah C.S., Potter G.B., Rubenstein J.L., Scheuer T., de la Iglesia H.O., Catterall W.A. (2012). Autistic-like behaviour in Scn1a+/− mice and rescue by enhanced GABA-mediated neurotransmission. Nature.

[18] Cui J., Park J., Ju X., Lee Y., Hong B., Ahn J., Kim Y.H., Ko Y., Yoon S.H., Lim C. (2021). General Anesthesia During Neurodevelopment Reduces Autistic Behavior in Adult BTBR Mice, a Murine Model of Autism. Front. Cell Neurosci..

[19] Meyza K.Z., Blanchard D.C. (2017). The BTBR mouse model of idiopathic autism—Current view on mechanisms. Neurosci. Biobehav. Rev..

[20] Garcia P.S., Kolesky S.E., Jenkins A. (2010). General anesthetic actions on GABA(A) receptors. Curr. Neuropharmacol..

[21] Franks N.P. (2008). General anaesthesia: From molecular targets to neuronal pathways of sleep and arousal. Nat. Rev. Neurosci..

[22] Hemmings H.C., Akabas M.H., Goldstein P.A., Trudell J.R., Orser B.A., Harrison N.L. (2005). Emerging molecular mechanisms of general anesthetic action. Trends Pharmacol. Sci..

[23] Li C., Schaefer M., Gray C., Yang Y., Furmanski O., Liu S., Worley P., Mintz C.D., Tao F., Johns R.A. (2017). Sensitivity to isoflurane anesthesia increases in autism spectrum disorder Shank3(+/c) mutant mouse model. Neurotoxicol. Teratol..

[24] Ichinose F., Mi W.-d., Miyazaki M., Onouchi T., Goto T., Morita S. (1998). Lack of Correlation between the Reduction of Sevoflurane MAC and the Cerebellar Cyclic GMP Concentrations in Mice Treated with 7-Nitroindazole. Anesthesiology.

[25] Lee S., Lee E., Kim R., Kim J., Lee S., Park H., Yang E., Kim H., Kim E. (2018). Shank2 Deletion in Parvalbumin Neurons Leads to Moderate Hyperactivity, Enhanced Self-Grooming and Suppressed Seizure Susceptibility in Mice. Front. Mol. Neurosci..

[26] Wang D., Huang Y., Wang X., Chen X., Li J., Zhang S., Wu J., Liu D., Ma D., Mei W. (2020). Circadian differences in emergence from volatile anaesthesia in mice: Involvement of the locus coeruleus noradrenergic system. Br. J. Anaesth..

[27] van den Brink I., van de Pol F., Vaneker M., Kox M., Schellekens W.J., Ritskes-Hoitinga M., Scheffer G.J. (2013). Mechanical ventilation of mice under general anesthesia during experimental procedures. Lab. Anim..

[28] Bokil H., Andrews P., Kulkarni J.E., Mehta S., Mitra P.P. (2010). Chronux: A platform for analyzing neural signals. J. Neurosci. Methods.

[29] Kenny J.D., Westover M.B., Ching S., Brown E.N., Solt K. (2014). Propofol and sevoflurane induce distinct burst suppression patterns in rats. Front. Syst. Neurosci..

[30] Wang D., Guo Q., Liu D., Kong X.-X., Xu Z., Zhou Y., Su Y., Dai F., Ding H.-L., Cao J.-L. (2021). Association Between Burst-Suppression Latency and Burst-Suppression Ratio Under Isoflurane or Adjuvant Drugs with Isoflurane Anesthesia in Mice. Front. Pharmacol..

[31] Baillet S., Friston K., Oostenveld R. (2011). Academic Software Applications for Electromagnetic Brain Mapping Using MEG and EEG. Comput. Intell. Neurosci..

[32] Han H.-B., Kim B., Kim Y., Jeong Y., Choi J.H. (2022). Nine-day continuous recording of EEG and 2-hour of high-density EEG under chronic sleep restriction in mice. Sci. Data.

[33] Pilge S., Jordan D., Kreuzer M., Kochs E.F., Schneider G. (2014). Burst suppression-MAC and burst suppression-CP_50_ as measures of cerebral effects of anaesthetics. Br. J. Anaesth..

[34] Jameson L.C., Sloan T.B. (2006). Using EEG to monitor anesthesia drug effects during surgery. J. Clin. Monit. Comput..

[35] Akeju O., Hamilos A.E., Song A.H., Pavone K.J., Purdon P.L., Brown E.N. (2016). GABAA circuit mechanisms are associated with ether anesthesia-induced unconsciousness. Clin. Neurophysiol..

[36] Pal D., Silverstein B.H., Sharba L., Li D., Hambrecht-Wiedbusch V.S., Hudetz A.G., Mashour G.A. (2017). Propofol, Sevoflurane, and Ketamine Induce a Reversible Increase in Delta-Gamma and Theta-Gamma Phase-Amplitude Coupling in Frontal Cortex of Rat. Front. Syst. Neurosci..

[37] Radovanovic L., Novakovic A., Petrovic J., Saponjic J. (2023). Different Alterations of Hippocampal and Reticulo-Thalamic GABAergic Parvalbumin-Expressing Interneurons Underlie Different States of Unconsciousness. Int. J. Mol. Sci..

[38] Hooshmandi M., Ho-Tieng D., Lister K.C., Cai W., Wong C., Brown N., Fan J., Hovhannisyan V., Uttam S., Prager-Khoutorsky M. (2025). Postnatal downregulation of Fmr1 in microglia promotes microglial reactivity and causes behavioural alterations in female mice. Mol. Autism.

[39] Montagni E., Ambrosone M., Martello A., Curti L., Polverini F., Baroncelli L., Mannaioni G., Pavone F.S., Masi A., Allegra Mascaro A.L. (2025). Age-dependent cortical overconnectivity in Shank3 mice is reversed by anesthesia. Transl. Psychiatry.

[40] Eger E.I., Saidman L.J., Brandstater B. (1965). Minimum alveolar anesthetic concentration: A standard of anesthetic potency. Anesthesiology.

[41] Rampil I.J., Mason P., Singh H. (1993). Anesthetic potency (MAC) is independent of forebrain structures in the rat. Anesthesiology.

[42] Sonner J.M., Antognini J.F., Dutton R.C., Flood P., Gray A.T., Harris R.A., Homanics G.E., Kendig J., Orser B., Raines D.E. (2003). Inhaled anesthetics and immobility: Mechanisms, mysteries, and minimum alveolar anesthetic concentration. Anesth. Analg..

[43] Ueyama H., Hagihira S., Takashina M., Nakae A., Mashimo T. (2010). Pregnancy does not enhance volatile anesthetic sensitivity on the brain: An electroencephalographic analysis study. Anesthesiology.

[44] Purdon P.L., Sampson A., Pavone K.J., Brown E.N. (2015). Clinical Electroencephalography for Anesthesiologists. Anesthesiology.

[45] Brown E.N., Lydic R., Schiff N.D. (2010). General Anesthesia, Sleep, and Coma. N. Engl. J. Med..

[46] Buzsáki G. (2002). Theta Oscillations in the Hippocampus. Neuron.

[47] Cavanagh J.F., Frank M.J. (2014). Frontal theta as a mechanism for cognitive control. Trends Cogn. Sci..

[48] Chander D., García P.S., Maccoll J.N., Illing S., Sleigh J.W. (2014). Electroencephalographic Variation during End Maintenance and Emergence from Surgical Anesthesia. PLoS ONE.

[49] Vijayan S., Ching S., Purdon P.L., Brown E.N., Kopell N.J. (2013). Thalamocortical Mechanisms for the Anteriorization of Alpha Rhythms during Propofol-Induced Unconsciousness. J. Neurosci..

[50] Wildes T.S., Mickle A.M., Ben Abdallah A., Maybrier H.R., Oberhaus J., Budelier T.P., Kronzer A., McKinnon S.L., Park D., Torres B.A. (2019). Effect of Electroencephalography-Guided Anesthetic Administration on Postoperative Delirium Among Older Adults Undergoing Major Surgery: The ENGAGES Randomized Clinical Trial. JAMA.

[51] Evered L.A., Chan M.T.V., Han R., Chu M.H.M., Cheng B.P., Scott D.A., Pryor K.O., Sessler D.I., Veselis R., Frampton C. (2021). Anaesthetic depth and delirium after major surgery: A randomised clinical trial. Br. J. Anaesth..

[52] Shinohe A., Hashimoto K., Nakamura K., Tsujii M., Iwata Y., Tsuchiya K.J., Sekine Y., Suda S., Suzuki K., Sugihara G. (2006). Increased serum levels of glutamate in adult patients with autism. Prog. Neuropsychopharmacol. Biol. Psychiatry.

